# Complex advance care planning intervention in general practice (ACP-GP): a cluster-randomised controlled trial

**DOI:** 10.3399/BJGP.2023.0022

**Published:** 2024-01-23

**Authors:** Julie Stevens, Rose Miranda, Peter Pype, Kim Eecloo, Luc Deliens, Aline De Vleminck, Koen Pardon

**Affiliations:** End-of-Life Care Research Group, VUB & Universiteit Gent, Brussels; Department of Family Medicine and Chronic Care, VUB, Brussels; Department of Public Health and Primary Care, Universiteit Gent, Ghent.; End-of-Life Care Research Group, VUB & Universiteit Gent, Brussels; Department of Family Medicine and Chronic Care, VUB, Brussels.; End-of-Life Care Research Group, VUB & Universiteit Gent, Brussels; Department of Public Health and Primary Care, Universiteit Gent, Ghent.; End-of-Life Care Research Group, VUB & Universiteit Gent, Brussels; Department of Public Health and Primary Care, Universiteit Gent, Ghent.; End-of-Life Care Research Group, VUB & Universiteit Gent, Brussels; Department of Family Medicine and Chronic Care, VUB, Brussels; Department of Public Health and Primary Care, Universiteit Gent, Ghent.; End-of-Life Care Research Group, VUB & Universiteit Gent, Brussels; Department of Family Medicine and Chronic Care, VUB, Brussels.; End-of-Life Care Research Group, VUB & Universiteit Gent, Brussels; Department of Family Medicine and Chronic Care, VUB, Brussels.

**Keywords:** advance care planning, chronic disease, randomised controlled trial

## Abstract

**Background:**

Advance care planning (ACP) is an iterative communication process about patients’ preferences for future care. In general practice, there are barriers to ACP at patient, GP, and healthcare-system levels. A complex intervention may be necessary to reduce barriers.

**Aim:**

To evaluate the effects of a complex ACP intervention for patients with chronic, life-limiting illness in general practice (ACP-GP).

**Design and setting:**

A cluster-randomised controlled trial was undertaken in Belgian general practice.

**Method:**

ACP-GP included a patient workbook, GP training, ACP conversations, and a documentation template. The control group received usual care. Outcomes were the 15-item ACP Engagement Survey for patients and the ACP Self-Efficacy scale for GPs. Linear mixed models evaluated differences at 3 months (T1, effectiveness evaluation) and 6 months (T2) post-baseline. Analysis was intention-to-treat.

**Results:**

In total, 35 GPs and 95 patients were randomised. Patient ACP engagement did not differ between the intervention and control group at T1 (baseline-adjusted mean difference = 0.34; 95% confidence interval [CI] = −0.02 to 0.69; *P* = 0.062) or T2 (baseline-adjusted mean difference = 0.20; 95% CI = −0.17 to 0.57; *P* = 0.28). For GP ACP self-efficacy, there were no significant differences between groups at T1 (baseline-adjusted mean difference = 0.16; 95% CI = −0.04 to 0.35; *P* = 0.11) or at T2 (baseline-adjusted mean difference = 0.11; 95% CI = −0.09 to 0.31; *P* = 0.27).

**Conclusion:**

ACP-GP did not improve patient engagement and GP self-efficacy more than usual care. Both groups showed patterns of increase from baseline. Trial procedures and the COVID-19 pandemic may have increased awareness about ACP.

## Introduction

In an ageing population, chronic life-limiting illnesses, such as cancer and cardiovascular disease, are prevalent causes of death.^1^ During exacerbations of these conditions, patients may face complex care choices or be unable to participate in medical decisions. Communicating preferences for care before exacerbation of the illness may ease decisional conflict for the patient and give patients a sense of control and peace of mind.^2^^,^^3^ For their family, it may reduce psychological distress and complicated grief.^4^

Advance care planning (ACP) is a process to facilitate communication about patient values, goals, and care preferences with health providers and loved ones.^5^ Recent conceptualisations of ACP emphasise the importance of an ongoing and iterative process that prepares patients and their surrogate decision makers to make better in-the-moment decisions about care.^6^ A longitudinal care setting with a trusting relationship, such as general practice, provides an environment for proactively encouraging patients to communicate, reflect on, and clarify their values over time.^7^^,^^8^

Research has shown that patients are willing to talk about ACP,^9^ but deficits have been found in its initiation.^10^ Barriers to ACP occur at different levels. For instance, patients may find ACP topics too emotional, uncomfortable, or not relevant. They might also lack knowledge about ACP, worry about the impact of ACP on relationships, or feel that the GP should initiate conversations.^9^^,^^11^^–^^14^ GPs may lack skills or confidence to discuss ACP, fear that ACP will deprive patients of hope, feel that patients should initiate conversations, or feel uncertain about timing.^15^^–^^17^ At the healthcare-system level, barriers include limited time and resources,^16^ and a lack of standard templates and mechanisms for sharing ACP.^17^

ACP intervention studies in general practice that target barriers at multiple levels remain scarce and disparate.^17^ Previous studies have recommended that communication training for GPs may address barriers related to perceived lack of skill or confidence.^18^^–^^20^ For patients, models based on behaviour change and social cognitive theories posit that processes, such as self-efficacy and readiness, underlie engagement in ACP. In these models, readiness to engage in ACP is an important precursor to patients taking action, such as by discussing care preferences.^21^^,^^22^ Educating patients about ACP and encouraging them to reflect on values and care wishes may promote engagement, helping them prepare for ACP discussions.^23^ The authors of the present study have previously also found that patients have greater ACP engagement overall, and greater ACP self-efficacy, when they rate highly the extent to which their GP listens to their worries about future health, emphasising the importance of communication.^24^ To address identified barriers and facilitate the initiation of ACP, a complex intervention for general practice (ACP-GP intervention) was developed and pilot-tested following the Medical Research Council framework.^23^^,^^25^^,^^26^ The present study aimed to evaluate the effects of the ACP-GP intervention on ACP engagement of patients with chronic, life-limiting illnesses and on GPs’ ACP self-efficacy.

**Table:** How this fits in

A complex intervention may be necessary to address barriers to advance care planning (ACP) within general practice. This study aimed to evaluate the effects of a complex ACP intervention for patients with chronic, life-limiting illnesses in general practice, on patient ACP engagement, and GP ACP self-efficacy. This study found no differences in outcome increases between the group receiving the ACP-GP intervention and the usual care control. GPs may feel confident in their skills to conduct ACP, and awareness of ACP and its relevance may already have an impact on patients thinking about, planning, and conducting ACP conversations.

## Method

### Design

A cluster-randomised controlled trial (RCT) was performed, with randomisation at the GP level to avoid contamination.^27^ Baseline data from this study have been analysed.^24^ To report this cluster-RCT, the Consolidated Standards of Reporting Trials (CONSORT) statement extension for cluster-randomised trials was used.^28^

### Setting and participants

Dutch-speaking GPs working in Flanders and Brussels, Belgium, were eligible for participation. In group settings, one GP per practice could participate. GPs identified patients for inclusion using an information card, which specified inclusion and exclusion criteria, shown in Box 1. Deviation from the protocol occurred to increase recruitment, by allowing GPs to participate if they could include at least one patient in the study, instead of three.

**Box 1.** Patient inclusion and exclusion criteria

**Inclusion criteria**	**Exclusion criteria**
Adults (aged >18 years)	Unable to speak or understand Dutch
Mentally competent as measured by judgement of the GP OR if Mini-Mental State Examination has been conducted, score is >24	Unable to provide consent or complete the questionnaires owing to cognitive impairment (as judged by the GP)
GP answers ‘no’ to surprise question: ‘Would I be surprised if this patient were to die within the next 12–24 months?’	GP answers ‘no’ to surprise question: ‘Would I be surprised if this patient were to die within the next 6 months?’
Diagnosis of a life-limiting illness: Locally advanced unresectable or metastasised cancer OR Organ failure, this being heart failure (New York Heart Association stage 3 or stage 4) chronic kidney failure or end-stage renal disease (stage 4, eGFR = 15–29; or stage 5, eGFR<15) Very severe COPD (GOLD COPD stages 3 or 4) OR Geriatric frailty (Clinical Frailty Scale score 5–7, mildly to severely frail)	Participated in the pilot study of this intervention or in the cognitive testing of the adjusted intervention materials
	Participating in other studies evaluating advance care planning, palliative care services, or communication strategies

*COPD = chronic obstructive pulmonary disease. eGFR = estimated glomerular filtration rate.*

### Intervention

Development of the intervention is reported elsewhere.^21^^,^^22^ Patients received the ACP-GP intervention for 6 months. Box 2 contains an overview of the intervention.

**Box 2.** ACP-GP intervention components

**Component**	**Description**
1. GP training	The ACP-GP training was initially developed as face-to-face training. It was adapted to an online format to accommodate COVID-19 pandemic restrictions in Belgium.
	Two interactive, small-group web sessions were provided by two trainers experienced in primary care and communication. Each session lasted approximately 2 hours. GPs received preparatory materials and background information through an e-learning module, which remained available throughout the course of the study. Intervention materials, such as the conversation guide and an example of the patient workbook, were made available in PDF format.
	In session 1, GPs discussed their experiences with ACP, fictional case examples and reflection questions, barriers to and facilitators for ACP, and video examples. In session 2, GPs practised intervention-specific ACP conversations with model patients, based on the patient workbook, followed by interactive feedback and discussion.

2. ACP workbook for patients	Patients received an ACP workbook (titled *My Wishes for Future Care*), which highlights the importance of ACP at different stages of health. Patients could use the workbook to reflect on topics such as quality of life, worries about future health or care, preferences for decision making, and whom they can ask to act as a surrogate decision maker.

3. Patient-centred ACP discussion with conversation guide	After the training, GPs were asked to conduct a minimum of two ACP conversations with each patient: conversation one within 2 weeks after the training, and conversation two within 1 month after the first conversation. The workbook for patients, and the ACP conversation guide for GPs, structured the conversation. GPs were reimbursed by the research team for the consultations.

4. Documentation of the ACP discussion	GPs received a documentation template, based on the conversation guide, which they could fill in to make notes of the outcomes of the ACP discussion.

*ACP = advance care planning.*

The control group received care as usual. GPs were not instructed to plan additional ACP conversations, but ACP could be spontaneously addressed during consultations.

### Data collection

Patients completed questionnaires on paper, with in-person or telephone assistance from independent data collectors if needed. GPs completed questionnaires via Qualtrics software or on paper. Patient and GP data were collected at baseline (month 0) and post-intervention measurements at 3 months and 6 months.

### Measures

Demographic information was self-reported via a questionnaire at baseline.

This paper reports the two separate primary outcomes of the trial, evaluated for effectiveness at 3-months’ follow up (T1) with exploratory comparison at T2.

The primary patient outcome was ACP engagement, measured using the ACP Engagement Survey 15-item version.^29^ Questions are on a 5-point Likert scale. The scale consists of the following two subscales: ACP self-efficacy (6 items) and ACP readiness (9 items). Overall engagement is the mean of all 15 items, where a higher score indicates greater engagement.

The primary GP outcome was self-efficacy, measured using the ACP Self-Efficacy (ACP-SE) scale, comprising 17 items plus one reference item on a 5-point Likert scale. The scale score is calculated as the average of the first 17 items; higher scores indicate greater self-efficacy. The reference item is a global single-item measure of self-efficacy, used for comparison with the scale.^30^

### Randomisation

GPs and their patients were allocated to intervention or control using a 1:1 ratio from a computer-generated list, with permuted block randomisation of varying block sizes. An independent statistician generated the list. GPs who gave informed consent, identified patients, and completed baseline assessments were allocated by an independent researcher to control or intervention.

Informed consent was sought from all participants. In contrast with the protocol, randomisation took place after GP consent, baseline, and identification of patients who could participate (before patient-informed consent and baseline assessment as originally planned), owing to timing constraints.

### Statistical methods

Sample-size estimates were conducted for outcomes at T1 at both patient and GP level, assuming equal cluster sizes of two patients and an intracluster correlation coefficient of 0.04.^31^ To achieve >90% power to detect mean differences of 1 at an alpha of 2.5% (Bonferroni correction), the study aimed to recruit 18 GPs per group, each with three patients (108 patients total), after accounting for dropout.

As distributions of patient age, GP age, and GP years of practice were skewed, sample median values and range were used to report these variables. Patient and GP outcomes were calculated as mean scale or subscale scores.

Linear mixed-model analyses were conducted with fixed effects of group, time, and group*time. Random intercepts in the models accounted for the clustered design (patients clustered within GPs, and measurements clustered within GPs and patients).

Estimated marginal means, baseline-adjusted mean differences, and their 95% confidence intervals (CIs) are reported. A *P*-value of 0.025 is used for scale scores at T1. Subscale scores and scores at T2 are interpreted at *P* = 0.05. Analysis was by intention-to-treat. All patients and GPs were included in the analysis in IBM SPSS Statistics (version 27).

## Results

### Recruitment and study flow

**Figure 1.**
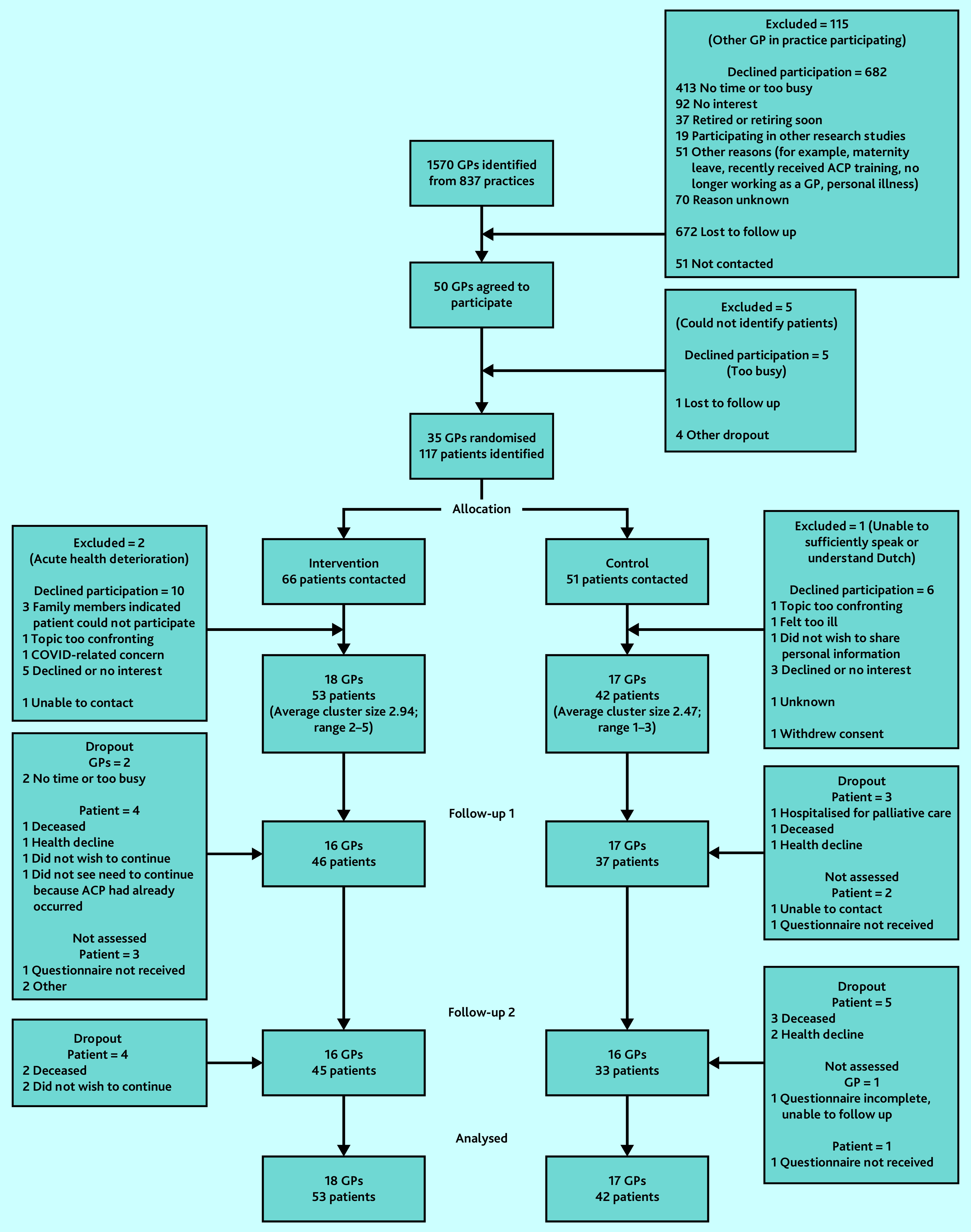
CONSORT flow diagram. Patients described as ‘not assessed’ at T1 (follow-up 1) were retained and approached again at T2 (follow-up 2) and were not considered dropouts.

Figure 1 shows recruitment, randomisation, and follow up. Owing to COVID-19 pandemic restrictions, the start of recruitment was postponed to June 2020. Inclusion of patients ended in December 2020.

Of 1570 GPs identified, 35 were randomised; 95 patients consented to participate. The final T2 questionnaires were returned in July 2021. The characteristics of patients and GPs are presented in Table 1.

**Table 1.** Participant characteristics by study arm

**Characteristic**	**Control, *n* (%)**	**Intervention, *n* (%)**
**Patients**	**42^a^**	**53**

Aged ≥80 years (sample median; sample range 42–95)	23 (54.8)	25 (47.2)

Female	25 (59.5)	25 (47.2)

**Marital status**		
Married, civil union, or domestic partnership	17 (40.5)	28 (52.8)
Widow(er)	17 (40.5)	20 (37.7)
Divorced, or single never married	8 (19.0)	5 (9.4)

**Highest educational attainment**		
Primary school	5 (11.9)	13 (24.5)
Secondary school	29 (69.0)	33 (62.3)
Post-secondary school	6 (14.3)	7 (13.2)
None of the above	2 (4.8)	0 (0.0)

**Person most involved in care**		
Spouse or partner	11 (26.8)	24 (45.3)
Child	17 (41.5)	15 (28.3)
Other family member	5 (12.2)	7 (13.2)
Other, not a family member	7 (17.1)	6 (11.3)
No person identified	1 (2.4)	1 (1.9)

Living together with person most involved in care	11 (27.5)	24 (45.3)

**Religion**		
Religious (Christianity)	26 (61.9)	31 (58.5)
Not religious	15 (35.7)	20 (37.7)
Prefer not to say	1 (2.4)	2 (3.8)

**Advance directives (AD) completed^b^**		
AD to refuse medical interventions	7 (16.7)	8 (15.1)
AD for euthanasia**^c^**	9 (21.4)	9 (17.0)
AD for funeral arrangements	5 (11.9)	4 (7.5)
AD for organ donation	1 (2.4)	3 (5.7)
Testament for donating the body to medical science after death	1 (2.4)	1 (1.9)
Other directive(s)	0 (0.0)	4 (7.5)
None	31 (73.8)	39 (73.6)

Oncological diagnosis	15 (35.7)	17 (32.1)

**GPs**	**17**	**18**

Aged ≥37 years (sample median; sample range 26–64)	6 (35.3)	12 (66.7)

Female	11 (64.7)	9 (50.0)

Years of practice experience ≥9 (sample median; sample range 1–39)	7 (41.2)	12 (66.7)

**Practice type^d^**		
Solo	4 (23.5)	4 (22.2)
Group	9 (52.9)	12 (66.7)
Primary care centre^e^	3 (17.6)	1 (5.6)
Hospital	0 (0.0)	0 (0.0)
Multiple	1 (5.9)	1 (5.6)

Coordinating and advisory physician^f^	3 (17.6)	1 (5.6)

Palliative home care team member	1 (5.9)	0 (0.0)

**Prior training in advance care planning**		
None	14 (82.4)	13 (72.2)
Introductory	2 (11.8)	5 (27.8)
Intensive	1 (5.9)	0 (0.0)

**Prior training in palliative care**		
None	11 (64.7)	11 (61.1)
Introductory	5 (29.4)	6 (33.3)
Intensive	1 (5.9)	1 (5.6)

a *Missing values: person most involved in care* n *= 1 and living together with person most involved in care* n *= 2.*

b *Multiple responses possible.*

c *AD for euthanasia in the case of irreversible coma.*

d *Belgian GPs are providers of primary care; GPs may work in single-physician (solo) practices, in (sometimes multidisciplinary) group practices with multiple GPs, and in multidisciplinary primary care centres.*

e *Primary care setting with a multidisciplinary collaboration, including ≥1 GPs, which is highly accessible and has a low financial threshold.*

f *GP, preferably trained in gerontology, who is responsible for the coordination, organisation, and continuity of medical care within a nursing home. A coordinating and advisory physician also manages the training of nursing home staff, including in the field of palliative care.*

The GP training, documentation template, and patient workbook were provided to the intervention group by the research team. At their respective T1 assessment, 13/16 GPs (81.25%) in the intervention group and 5/17 GPs (29.41%) in the control group reported having had ACP conversations with patients included in the study. In the intervention group, 33/46 patients (71.74%) reported at least one ACP conversation with their GP at T1; 14 (30.43%) reported ≥2 conversations. In the control group, 12/37 patients (32.43%) reported having at least one ACP conversation, with six (16.22%) reporting ≥2 (data not shown).

### Patient outcomes

There was no significant difference in patient ACP engagement between intervention and control groups at 3 months post-baseline (baseline-adjusted mean difference = 0.34; 95% CI = −0.02 to 0.69; *P* = 0.062; standardised effect size = 0.34) nor at 6 months post-baseline (baseline-adjusted mean difference = 0.20; 95% CI = −0.17 to 0.57; *P* = 0.28; standardised effect size = 0.20; Table 2). Strikingly, patterns of increasing ACP engagement in both groups were found from baseline to month 3, and baseline to month 6. Similar increasing patterns from baseline versus month 3 and 6 were observed in the subscales for ACP self-efficacy and ACP readiness for patients in both groups.

**Table 2.** Patient outcome: cluster-adjusted mean scores and differences for ACP engagement^a^

	**Baseline (T0)**		**T1 (3 months)**					**T2 (6 months)**					
	**EMM (95% CI)**		**EMM (95% CI)**		**Baseline- adjusted mean difference (95% CI)**	***P* -value**	**Effect size (standardised)**	**EMM (95% CI)**		**Baseline- adjusted mean difference (95% CI)**	***P*-value**	**Effect size (standardised)**	**Intra-class correlation coefficient**
	**Control**	**Intervention**	**Control**	**Intervention**				**Control**	**Intervention**				
**ACP engagement overall**	**3.02 (2.72 to 3.33)**	**3.06 (2.79 to 3.33)**	**3.40 (3.09 to 3.71)**	**3.77 (3.49 to 4.06)**	**0.34 (−0.02 to 0.69)**	**0.062**	**0.34**	**3.69 (3.37 to 4.01)**	**3.93 (3.64 to 4.22)**	**0.20 (−0.17 to 0.57)**	**0.28**	0.20	0.043
ACP self-efficacy	3.81 (3.50 to 4.11)	3.91 (3.64 to 4.18)	3.88 (3.56 to 4.20)	4.25 (3.95 to 4.55)	0.26 (−0.15 to 0.68)	0.22	0.27	4.06 (3.73 to 4.39)	4.25 (3.96 to 4.55)	0.09 (−0.33 to 0.51)	0.67	0.09	0.01
ACP readiness	2.52 (2.16 to 2.90)	2.48 (2.14 to 2.82)	3.07 (2.68 to 3.46)	3.43 (3.07 to 3.78)	0.40 (−0.06 to 0.86)	0.088	0.32	3.45 (3.05 to 3.85)	3.69 (3.33 to 4.06)	0.29 (−0.18 to 0.76)	0.23	0.23	0.001

a ACP Engagement Survey 15-item version consists of 15 items on a 5-point (1–5) Likert scale. Self-efficacy subscale = 6 items; readiness subscale = 9 items. Overall ACP engagement is the mean of all items. Self-efficacy and readiness subscale scores are the mean of all items within the subscale. If <25% of data were missing for a respective scale or subscale, the mean was computed of the answered items. If >25% of data were missing, the mean was coded as missing. Higher scores indicate greater overall engagement, self-efficacy, or readiness. ACP self-efficacy range: 1 (not at all confident) to 5 (very confident). ACP readiness range: 1 (I have never thought about it) to 5 (I have already done it). Intra-class correlation coefficient for patients was calculated by applying a null model, with clustering within GPs, to baseline data. Standardised effect sizes were calculated by dividing the group*time coefficient by the standard deviation (square root of the summed linear mixed-model variance components). ACP = advance care planning. EMM = estimated marginal means.

### GP outcomes

GP ACP self-efficacy did not differ significantly between intervention and control groups at 3 months post-baseline (baseline-adjusted mean difference = 0.16; 95% CI = −0.04 to 0.35; *P* = 0.11; standardised effect size = 0.44), nor at 6 months post-baseline (baseline-adjusted mean difference = 0.11; 95% CI = −0.09 to 0.31; *P* = 0.27; standardised effect size = 0.31; Table 3). ACP self-efficacy was higher at month 3 and month 6 versus baseline, in both groups.

**Table 3.** GP outcome: cluster-adjusted mean scores and differences for ACP self-efficacy^a^

	**Baseline**		**T1 (3 months)**					**T2 (6 months)**				
	**EMM (95% CI)**		**EMM (95% CI)**		**Baseline- adjusted mean difference (95% CI)**	***P* -value**	**Effect size (standardised)**	**EMM (95% CI)**		**Baseline- adjusted mean difference (95% CI)**	***P*-value**	**Effect size (standardised)**
	**Control**	**Intervention**	**Control**	**Intervention**				**Control**	**Intervention**			
ACP Self- Efficacy (ACP-SE)	**3.81 (3.64 to 3.98)**	**3.83 (3.66 to 3.99)**	**3.95 (3.78 to 4.12)**	**4.12 (3.95 to 4.29)**	**0.16 (−0.04 to 0.35)**	**0.11**	**0.44**	**3.99 (3.82 to 4.16)**	**4.11 (3.94 to 4.28)**	**0.11 (−0.09 to 0.31)**	**0.27**	**0.31**
Reference item ACP-SE 18 (How confident are you that you can engage patients in ACP conversations?)	3.82 (3.57 to 4.08)	3.83 (3.59 to 4.08)	4.00 (3.75 to 4.26)	3.88 (3.62 to 4.14)	−0.13 (−0.52 to 0.26)	0.52	−0.24	3.86 (3.60 to 4.12)	4.01 (3.75 to 4.27)	0.14 (−0.25 to 0.53)	0.48	0.26

a The ACP Self-Efficacy (ACP-SE) scale consists of 18 items on a 5-point Likert scale. ACP-SE scale score is the mean of the first 17 items. Item 18 is a reference item for comparison. A higher score indicates higher self-efficacy. ACP-SE range: 1 (I know with certainty that I CANNOT do it) to 5 (I know with certainty that I CAN do it). Standardised effect sizes were calculated by dividing the group*time coefficient by the standard deviation (square root of the summed linear mixed-model variance components). ACP = advance care planning. EMM = estimated marginal means.

## Discussion

### Summary

A cluster-RCT was conducted of a complex ACP intervention for patients with chronic, life-limiting illnesses in general practice. No differences were found in the improvement of patient ACP engagement or GP ACP self-efficacy between the group assigned to the ACP-GP intervention, and the group assigned to usual care. However, the study found increases in the overall patients’ ACP engagement, including the subscales ACP self-efficacy and readiness, and the GPs’ self-efficacy during the 6 months of observation in both the intervention and control groups.

### Strengths and limitations

This study has several strengths. The ACP-GP intervention was robustly developed and pilot-tested,^23^^,^^25^ according to the widely accepted Medical Research Council framework,^26^^,^^32^ which combines structured and iterative steps to evaluate complex interventions while reflecting on intervention context and theory. Additionally, validated instruments were used, which aimed to investigate behaviour-change processes underlying ACP actions.^22^

This study also had limitations. As the trial occurred during the COVID-19 pandemic, GPs reported extraordinary time and workload pressures, and difficulty identifying eligible patients. Additionally, allowing GPs to identify patients for inclusion may have introduced selection bias towards patients the GP judged to be more amenable to ACP, or with whom the GP felt were more comfortable discussing ACP. This choice of recruitment design was made to minimise risks of interfering with the existing GP–patient relationship.

### Comparison with existing literature

Several reasons can explain why this intervention did not reach its intended outcomes. First, patient ACP engagement and GP self-efficacy showed increases from baseline to 3 months and 6 months in both the intervention and control groups. Although ACP conversations were possible as part of usual care, the authors expected few to take place. However, GPs in both groups reported ACP conversations, as did 12 patients in the control group. Hearing about ACP through the informed consent procedures, and answering the questionnaire, may have made patients and GPs, including those in the control group, aware of ACP. This may have activated both patients and GPs in the control group to prepare for or conduct ACP discussions more than expected. A 2016 cluster-RCT has similarly suggested that an intervention creating awareness of optimal symptom relief in dementia may be more effective than a physician practice guideline.^33^ More recently, a cluster-RCT of a complex ACP intervention has proposed similar awareness-raising across groups as a result of study procedures, or a Hawthorne effect.^34^

Second, emergent literature on the impact of the COVID-19 pandemic on ACP^35^ may frame this finding, as the study period overlapped with the first, second, and third waves of the pandemic in Belgium.^36^^–^^38^ A Belgian survey found worries among the general population about their current health state and their access to health care during the first 8 weeks of lockdown, including in the highest age bracket (≥66 years).^39^ It is possible that these concerns persisted during subsequent waves and periods of lockdown. Concerns about COVID-19 in patients with vulnerable health may have encouraged patients to think about and/or discuss end-of-life issues and ACP, regardless of group.

Owing to COVID-19, the implementation of the intervention may also not have been optimal. In Belgium, triage-and-testing centres were established to reduce the risk of spreading COVID-19 and to screen (a) symptomatic individuals. Coordination of these centres was entrusted to regional GP groups.^40^ GPs were advised to give priority to patients showing symptoms of COVID-19, and to maintain the continuity of non-COVID-19-related care. GP practices were permitted to adopt means including systems of (telephone) triage, reserved time slots for priority and non-priority groups, and appointment systems. Nevertheless, GPs expressed that, during the first wave of COVID-19 in Belgium, chronic care activities often lessened.^41^ Even before the pandemic, difficulties for GPs to fully engage in studies in palliative care have been documented.^42^ Owing to COVID-19 restrictions, rather than in-person training, the GP training was delivered online. Evidence has suggested that online training can be as effective as in-person,^43^^,^^44^ and online training in serious illness communication for intensive care unit (ICU) nurses was effective and acceptable.^45^ Nevertheless, more research may be needed to assess its implementation in continuing medical education for GPs specifically. Moreover, GPs may need more time to consolidate and practise what they have learnt, as has been suggested for care staff in a complex ACP intervention in nursing homes.^46^

Third, recent research has increasingly highlighted the importance of ACP processes such as readiness. It is possible that, while patients feel relatively confident that they can discuss ACP, readiness remains variable.^47^^,^^48^ A scoping review found significant effects in three studies in primary care clinics that measured the ACP Engagement Survey in the US.^4^ The studies used the PREPARE For Your Care programme, which includes a website to motivate and prepare patients for ACP conversations, as well as an easy-to-read advance directive provided to both study arms.^49^ Compared with a 2022 study of a web-based ACP programme in the Netherlands, using the 34-item Dutch ACP Engagement Survey, the authors of the present study found that readiness for ACP especially appeared to increase more in both ACP-GP study groups.^50^ A trial of an interactive ACP guide, *Plan Well Guide*, for patients at high risk of health decline showed an increase in both groups, and potentially larger increases in readiness than self-efficacy,^51^ similar to findings in the current trial.

Finally, ACP self-efficacy in GPs merits reflection. In the present study, self-efficacy was relatively high at baseline, which may impose ceiling effects on the outcome at follow up. Primary care professionals may have more self-efficacy if they feel sufficiently trained.^52^ However, in a review of end-of-life communication interventions, training for health providers showed mixed effects on confidence.^53^ Despite literature suggesting a lack of self-efficacy or confidence may be a GP-level barrier, recent studies have found high willingness and confidence for ACP in Canadian primary care providers. However, engagement in ACP remained low.^19^^,^^54^

### Implications for research and practice

While the ACP-GP intervention did not improve patients’ ACP engagement and GPs’ self-efficacy, results of this trial have contributed important insights to the field of ACP research, which has seen intensive reflection regarding future directions.^55^^,^^56^

Patterns of increasing ACP engagement were seen in the intervention group and the usual-care control. The design and context of the trial, including questionnaires that explain ACP, as well as the COVID-19 pandemic, which brought media attention and public awareness to ACP, may mean that the intervention was compared with an awareness condition or even a (community-based) intervention. This possible ‘shift in mindset’^57^ has highlighted the potential for a public health and media-messaging approach, which can help normalise ACP.^58^

Stakeholders consulted during the development of ACP-GP were mainly health providers. While this provided a depth of insight into GPs’ needs, it will be necessary to involve patient and surrogate decision makers more closely in the future, to ensure intervention components also fully match their expressed needs. Inviting patients to engage in ACP conversations, even with an accompanying workbook, may be insufficient if attitudes, emotional barriers, and social context are not addressed. Closer involvement of family or surrogate decision makers may be necessary to facilitate engagement, as some patients may also want informal discussions with family.^57^^,^^17^

The ACP-GP intervention is a complex intervention with multiple components targeting GPs and patients. The inherent complexity of ACP, involving multiple behaviours and participants,^4^ and the complexity of barriers to ACP, requires that interventions to facilitate ACP should account for this complexity by offering interacting components such as documentation and communication.^59^ While complexity does not necessarily equate to time-consuming or difficult interventions, it is nevertheless crucial to take into account increasing time and resource demands of the GP setting. For instance, if awareness-raising contributed to patient ACP engagement in both groups, the added value of the larger intervention should be carefully considered. In practice, ACP communication is more than a discrete number of appointments; it requires GPs to be aware of the wishes and concerns of patients and to be open to discussing these when the opportunity arises naturally.^60^

Considering the primary outcome findings in this trial, it is thus important to evaluate which components were (not) of perceived benefit to GPs and patients, how demanding the intervention was of time and resources, and how the components worked when implemented in the GP setting. An important next step will be a thorough process evaluation of the trial, where patients and GPs are invited to reflect on their experiences with the intervention. This will help identify how and why each component worked, and the challenges and facilitators encountered during implementation. The current study and the planned process evaluation of ACP-GP can contribute to insights regarding which components are effective and efficient.

## Data

The dataset analysed for this manuscript is available from the corresponding author on reasonable request.
